# 5-(Pyridin-4-yl)isophthalic acid

**DOI:** 10.1107/S1600536810039474

**Published:** 2010-10-23

**Authors:** Yong-Fang Zhang, Qing-Fu Zhang, Juan Jin, De-Zhi Sun, Da-Qi Wang

**Affiliations:** a College of Chemistry and Chemical Engineering, Liaocheng University, Shandong 252059, People’s Republic of China

## Abstract

In the title compound, C_13_H_9_NO_4_, the two carb­oxy­lic groups and the benzene ring are approximately co-planar with a maximum atomic deviation 0.175 (4) Å, while the pyridine ring is oriented at a dihedral angle of 31.07 (18)° with respect to the benzene ring. In the crystal, mol­ecules are linked by O—H⋯O, O—H⋯N and weak C—H⋯O hydrogen bonds, forming a three-dimensional supra­molecular framework.

## Related literature

For background to carb­oxy­lic acids as supra­molecular synthons, see: Desiraju (1995 ▶); Thalladi *et al.* (1996 ▶).
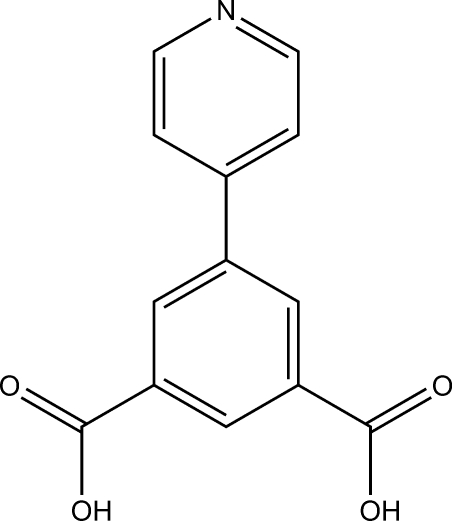


## Experimental

### 

#### Crystal data

C_13_H_9_NO_4_
*M* *_r_* = 243.21
Orthorhombic, 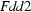

*a* = 15.5362 (16) Å
*b* = 37.371 (3) Å
*c* = 7.1716 (9) Å
*V* = 4163.9 (8) Å^3^
*Z* = 16
Mo *K*α radiation
μ = 0.12 mm^−1^
*T* = 296 K
0.30 × 0.20 × 0.10 mm

#### Data collection

Siemens SMART 1000 CCD area-detector diffractometer
Absorption correction: multi-scan (*SADABS*; Sheldrick, 1996 ▶) *T*_min_ = 0.966, *T*_max_ = 0.988
4224 measured reflections
1004 independent reflections
879 reflections with *I* > 2σ(*I*)
*R*_int_ = 0.045

#### Refinement

*R*[*F* ^2^ > 2σ(*F* ^2^)] = 0.039
*wR*(*F* ^2^) = 0.107
*S* = 1.04
1004 reflections
164 parameters
1 restraint
H-atom parameters constrained
Δρ_max_ = 0.33 e Å^−3^
Δρ_min_ = −0.21 e Å^−3^

### 

Data collection: *SMART* (Siemens, 1996 ▶); cell refinement: *SAINT* (Siemens, 1996 ▶); data reduction: *SAINT*; program(s) used to solve structure: *SHELXTL* (Sheldrick, 2008 ▶); program(s) used to refine structure: *SHELXTL*; molecular graphics: *SHELXTL*; software used to prepare material for publication: *SHELXTL*.

## Supplementary Material

Crystal structure: contains datablocks I, global. DOI: 10.1107/S1600536810039474/xu5043sup1.cif

Structure factors: contains datablocks I. DOI: 10.1107/S1600536810039474/xu5043Isup2.hkl

Additional supplementary materials:  crystallographic information; 3D view; checkCIF report

## Figures and Tables

**Table 1:** Hydrogen-bond geometry (Å, °)

*D*—H⋯*A*	*D*—H	H⋯*A*	*D*⋯*A*	*D*—H⋯*A*
O2—H2*A*⋯N1^i^	0.82	1.78	2.558 (3)	157
O3—H3*A*⋯O2^ii^	0.82	1.91	2.620 (3)	144
C10—H10⋯O1^iii^	0.93	2.57	3.491 (4)	172

Symmetry codes: (i) 
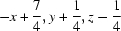
; (ii) 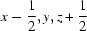; (iii) 
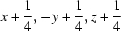
.

**Fig. 1.**
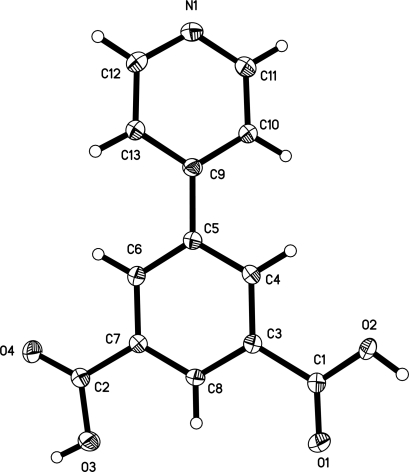
The molecular structure of the title compound, showing 30% probability displacement ellipsoids.

**Fig. 2.**
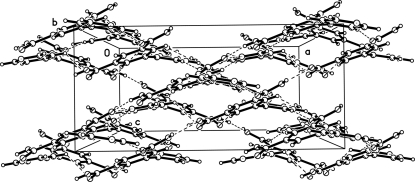
The packing diagram of the title compound.

## supplementary crystallographic information

### Comment

Carboxylic acid is an interesting supramolecular synthon, widely used to construct supramolecular array with one to three different dimensions *via* hydrogen bonds (Desiraju, 1995; Thalladi *et al.*, 1996). In order to explore this area, the structure of the title compound, 5-(pyridin-4-yl)isophthalic acid, is reported herein.

As shown in Fig.1, the two carboxylic groups and the phenyl ring system in the title compound are almost planar with the maximum deviation 0.175Å for atom O_2_ in the carboxylic group. However, the pyridine ring and the phenyl ring is not coplanar, with the dihedral angle 31.07°. This may be due to intermolecular O—H···N hydrogen-bonding interactions.

In the crystal structure, the dicarboxylic acid molecules are linked by intermolecular O—H···N, O—H···O and C—H···O hydrogen bonding interactions into a three-dimensional framework (Fig. 2).

### Experimental

The commercially available title compound, 5-(pyridin-4-yl)isophthalic acid, was recrystallized from an aqueous solution.

### Refinement

All H atoms were positioned geometrically and constrained to ride on their parent atoms with C—H = 0.93 Å and *U*_iso_(H) = 1.2*U*_eq_(C), O—H = 0.82 Å and *U*_iso_(H) = 1.5*U*_eq_(O). As no significant anomalous scatterings, Friedel pairs were merged.

### Crystal data

**Table:**

C_13_H_9_NO_4_	*F*(000) = 2016
*M**_r_* = 243.21	*D*_x_ = 1.552 Mg m^−^^3^
Orthorhombic, *F**d**d*2	Mo *K*α radiation, λ = 0.71073 Å
Hall symbol: F 2 -2d	Cell parameters from 1601 reflections
*a* = 15.5362 (16) Å	θ = 2.8–26.5°
*b* = 37.371 (3) Å	µ = 0.12 mm^−^^1^
*c* = 7.1716 (9) Å	*T* = 296 K
*V* = 4163.9 (8) Å^3^	Block, colorless
*Z* = 16	0.30 × 0.20 × 0.10 mm

### Data collection

**Table:**

Siemens SMART 1000 CCD area-detector diffractometer	1004 independent reflections
Radiation source: fine-focus sealed tube	879 reflections with *I* > 2σ(*I*)
graphite	*R*_int_ = 0.045
φ and ω scans	θ_max_ = 25.0°, θ_min_ = 2.2°
Absorption correction: multi-scan (*SADABS*; Sheldrick, 1996)	*h* = −18→8
*T*_min_ = 0.966, *T*_max_ = 0.988	*k* = −44→38
4224 measured reflections	*l* = −8→8

### Refinement

**Table:**

Refinement on *F*^2^	Secondary atom site location: difference Fourier map
Least-squares matrix: full	Hydrogen site location: inferred from neighbouring sites
*R*[*F*^2^ > 2σ(*F*^2^)] = 0.039	H-atom parameters constrained
*wR*(*F*^2^) = 0.107	*w* = 1/[σ^2^(*F*_o_^2^) + (0.0701*P*)^2^ + 2.9125*P*] where *P* = (*F*_o_^2^ + 2*F*_c_^2^)/3
*S* = 1.04	(Δ/σ)_max_ < 0.001
1004 reflections	Δρ_max_ = 0.33 e Å^−^^3^
164 parameters	Δρ_min_ = −0.21 e Å^−^^3^
1 restraint	Extinction correction: *SHELXL*, Fc^*^=kFc[1+0.001xFc^2^λ^3^/sin(2θ)]^-1/4^
Primary atom site location: structure-invariant direct methods	Extinction coefficient: 0.0010 (3)

### Special details

**Table:**

Geometry. All e.s.d.'s (except the e.s.d. in the dihedral angle between two l.s. planes) are estimated using the full covariance matrix. The cell e.s.d.'s are taken into account individually in the estimation of e.s.d.'s in distances, angles and torsion angles; correlations between e.s.d.'s in cell parameters are only used when they are defined by crystal symmetry. An approximate (isotropic) treatment of cell e.s.d.'s is used for estimating e.s.d.'s involving l.s. planes.
Refinement. Refinement of *F*^2^ against ALL reflections. The weighted *R*-factor *wR* and goodness of fit *S* are based on *F*^2^, conventional *R*-factors *R* are based on *F*, with *F* set to zero for negative *F*^2^. The threshold expression of *F*^2^ > σ(*F*^2^) is used only for calculating *R*-factors(gt) *etc*. and is not relevant to the choice of reflections for refinement. *R*-factors based on *F*^2^ are statistically about twice as large as those based on *F*, and *R*- factors based on ALL data will be even larger.

### Fractional atomic coordinates and isotropic or equivalent isotropic displacement parameters (Å^2^)

**Table:**

	*x*	*y*	*z*	*U*_iso_*/*U*_eq_	
O1	0.70913 (15)	0.16593 (6)	0.0729 (5)	0.0455 (7)	
O2	0.81725 (15)	0.13320 (6)	−0.0349 (5)	0.0401 (7)	
H2A	0.8354	0.1528	−0.0678	0.060*	
O3	0.45574 (15)	0.10618 (6)	0.3114 (4)	0.0450 (8)	
H3A	0.4068	0.1054	0.3541	0.068*	
O4	0.45297 (14)	0.04779 (6)	0.3757 (4)	0.0451 (8)	
N1	0.85168 (17)	−0.05737 (7)	0.1930 (5)	0.0351 (7)	
C1	0.74454 (19)	0.13706 (8)	0.0469 (5)	0.0290 (8)	
C2	0.4902 (2)	0.07358 (8)	0.3152 (6)	0.0294 (8)	
C3	0.70358 (19)	0.10312 (8)	0.1149 (5)	0.0265 (7)	
C4	0.74860 (19)	0.07115 (8)	0.1188 (5)	0.0274 (8)	
H4	0.8058	0.0707	0.0807	0.033*	
C5	0.70940 (18)	0.03966 (8)	0.1790 (5)	0.0257 (7)	
C6	0.62393 (19)	0.04072 (8)	0.2376 (5)	0.0283 (8)	
H6	0.5966	0.0198	0.2755	0.034*	
C7	0.57949 (19)	0.07292 (8)	0.2395 (5)	0.0270 (8)	
C8	0.6192 (2)	0.10389 (8)	0.1779 (5)	0.0268 (8)	
H8	0.5891	0.1254	0.1788	0.032*	
C9	0.7591 (2)	0.00577 (8)	0.1821 (5)	0.0276 (8)	
C10	0.8478 (2)	0.00577 (8)	0.2122 (6)	0.0330 (9)	
H10	0.8772	0.0272	0.2282	0.040*	
C11	0.8915 (2)	−0.02631 (9)	0.2179 (6)	0.0374 (9)	
H11	0.9505	−0.0262	0.2399	0.045*	
C12	0.7669 (2)	−0.05810 (9)	0.1627 (6)	0.0361 (9)	
H12	0.7397	−0.0800	0.1450	0.043*	
C13	0.7191 (2)	−0.02726 (8)	0.1570 (5)	0.0319 (8)	
H13	0.6601	−0.0284	0.1364	0.038*	

### Atomic displacement parameters (Å^2^)

**Table:**

	*U*^11^	*U*^22^	*U*^33^	*U*^12^	*U*^13^	*U*^23^
O1	0.0389 (14)	0.0232 (12)	0.074 (2)	0.0034 (10)	0.0125 (14)	0.0062 (14)
O2	0.0330 (12)	0.0211 (11)	0.0664 (18)	−0.0048 (10)	0.0166 (13)	0.0045 (11)
O3	0.0301 (12)	0.0342 (13)	0.071 (2)	0.0072 (10)	0.0199 (13)	0.0063 (14)
O4	0.0318 (13)	0.0342 (13)	0.069 (2)	−0.0040 (10)	0.0164 (13)	0.0055 (13)
N1	0.0337 (15)	0.0266 (14)	0.045 (2)	0.0059 (12)	0.0011 (14)	0.0036 (14)
C1	0.0239 (15)	0.0255 (17)	0.037 (2)	−0.0015 (13)	0.0017 (14)	0.0042 (15)
C2	0.0261 (15)	0.0287 (17)	0.033 (2)	−0.0007 (13)	0.0011 (15)	0.0030 (15)
C3	0.0240 (15)	0.0243 (15)	0.031 (2)	−0.0011 (12)	0.0009 (14)	0.0007 (14)
C4	0.0202 (15)	0.0279 (16)	0.034 (2)	0.0013 (12)	0.0025 (14)	−0.0003 (14)
C5	0.0241 (15)	0.0239 (15)	0.0289 (18)	0.0014 (12)	0.0013 (14)	0.0020 (15)
C6	0.0255 (16)	0.0251 (16)	0.034 (2)	−0.0024 (13)	0.0025 (15)	0.0008 (14)
C7	0.0238 (15)	0.0254 (16)	0.032 (2)	0.0003 (12)	−0.0006 (15)	0.0006 (14)
C8	0.0233 (15)	0.0234 (15)	0.034 (2)	0.0032 (12)	0.0020 (14)	0.0008 (15)
C9	0.0275 (16)	0.0253 (16)	0.030 (2)	0.0031 (13)	0.0064 (14)	0.0026 (15)
C10	0.0252 (15)	0.0253 (16)	0.048 (2)	−0.0004 (12)	0.0006 (16)	0.0026 (16)
C11	0.0269 (17)	0.0345 (19)	0.051 (3)	0.0037 (14)	−0.0009 (17)	0.0042 (17)
C12	0.0347 (18)	0.0246 (16)	0.049 (3)	−0.0007 (13)	0.0011 (18)	0.0024 (16)
C13	0.0277 (16)	0.0259 (15)	0.042 (2)	−0.0008 (13)	0.0018 (16)	0.0041 (15)

### Geometric parameters (Å, °)

**Table:**

O1—C1	1.225 (4)	C5—C6	1.393 (4)
O2—C1	1.281 (4)	C5—C9	1.483 (4)
O2—H2A	0.8200	C6—C7	1.387 (4)
O3—C2	1.331 (4)	C6—H6	0.9300
O3—H3A	0.8200	C7—C8	1.384 (4)
O4—C2	1.205 (4)	C8—H8	0.9300
N1—C11	1.327 (4)	C9—C10	1.395 (4)
N1—C12	1.335 (4)	C9—C13	1.394 (5)
C1—C3	1.501 (4)	C10—C11	1.378 (5)
C2—C7	1.489 (4)	C10—H10	0.9300
C3—C4	1.385 (4)	C11—H11	0.9300
C3—C8	1.386 (4)	C12—C13	1.372 (5)
C4—C5	1.394 (4)	C12—H12	0.9300
C4—H4	0.9300	C13—H13	0.9300
			
C1—O2—H2A	109.5	C8—C7—C6	120.0 (3)
C2—O3—H3A	109.5	C8—C7—C2	121.2 (3)
C11—N1—C12	120.0 (3)	C6—C7—C2	118.8 (3)
O1—C1—O2	124.4 (3)	C7—C8—C3	120.6 (3)
O1—C1—C3	120.3 (3)	C7—C8—H8	119.7
O2—C1—C3	115.3 (3)	C3—C8—H8	119.7
O4—C2—O3	123.1 (3)	C10—C9—C13	117.4 (3)
O4—C2—C7	124.4 (3)	C10—C9—C5	121.1 (3)
O3—C2—C7	112.5 (3)	C13—C9—C5	121.5 (3)
C4—C3—C8	119.3 (3)	C11—C10—C9	119.4 (3)
C4—C3—C1	121.4 (3)	C11—C10—H10	120.3
C8—C3—C1	119.3 (3)	C9—C10—H10	120.3
C3—C4—C5	120.9 (3)	N1—C11—C10	121.8 (3)
C3—C4—H4	119.5	N1—C11—H11	119.1
C5—C4—H4	119.5	C10—C11—H11	119.1
C6—C5—C4	119.1 (3)	N1—C12—C13	121.5 (3)
C6—C5—C9	121.1 (3)	N1—C12—H12	119.3
C4—C5—C9	119.9 (3)	C13—C12—H12	119.3
C7—C6—C5	120.2 (3)	C12—C13—C9	119.9 (3)
C7—C6—H6	119.9	C12—C13—H13	120.0
C5—C6—H6	119.9	C9—C13—H13	120.0

### Hydrogen-bond geometry (Å, °)

**Table:**

*D*—H···*A*	*D*—H	H···*A*	*D*···*A*	*D*—H···*A*
O2—H2A···N1^i^	0.82	1.78	2.558 (3)	157
O3—H3A···O2^ii^	0.82	1.91	2.620 (3)	144
C10—H10···O1^iii^	0.93	2.57	3.491 (4)	172

Symmetry codes: (i) −*x*+7/4, *y*+1/4, *z*−1/4; (ii) *x*−1/2, *y*, *z*+1/2; (iii) *x*+1/4, −*y*+1/4, *z*+1/4.
